# Improving Millennial Employees’ OCB: A Multilevel Mediated and Moderated Model of Ethical Leadership

**DOI:** 10.3390/ijerph18158139

**Published:** 2021-07-31

**Authors:** Wei Su, Juhee Hahn

**Affiliations:** 1 The Graduate School, Chung-Ang University, Seoul 06974, Korea; suv4591@gmail.com; 2 Department of Business Management, Chung-Ang University, Seoul 06974, Korea

**Keywords:** ethical leadership, ethical climate, affective well-being, moral identity, organizational citizenship behavior, millennials

## Abstract

In the field of organizational behavior, the influence of leadership in organizations and the organizational citizenship behavior (OCB) of employees have always been two hot topics studied by scholars. However, previous studies have mainly examined the OCB of baby boomers and Generation Xers. With millennials now entering the workforce, they will highly likely not take the initiative to engage in OCB due to their different values. Scholars have found that millennials respond well to ethical leadership. Although this statement has a theoretical basis, empirical research regarding this topic is still insufficient. Thus, this study explores whether ethical leadership can effectively promote millennials’ OCB. Moreover, the mediating effect of group-level ethical climate and individual-level affective well-being, and the moderating effect of individual-level moral identity, were examined. The study hypotheses were verified based on 384 valid questionnaires collected from 61 teams using Mplus 8.3. The results showed that (1) ethical leadership was a positive predictor of millennials’ OCB; (2) ethical climate and affective well-being partially mediated the relationship between ethical leadership and OCB; and (3) moral identity moderated the relationship between ethical leadership and affective well-being and the indirect impact of ethical leadership on OCB. These findings provide empirical support for applying social learning theory, social information processing theory, and conservation of resources (COR)theory. This research also provides several managerial implications through which managers can more effectively improve the OCB of millennial employees.

## 1. Introduction

By 2025, millennial workers will account for 75% of the global workforce, which means that three out of four workers will be millennials [1]. Although no precise birth years have been specified for millennials, the term “millennials” generally refers to those born between 1980 and 2000 [2]. Due to the unique values, expectations, and attitudes held by millennials, as compared to previous generations, this generational shift in the workforce will create opportunities and challenges for companies [3,4]. According to previous studies, the work values of millennials and the previous generations differ in four ways: (1) millennials pay more attention to extrinsic value [5]; (2) millennials have a stronger desire to obtain money and status from their work and are more concerned about leisure time [6,7]; (3) millennials tend to have lower social values, indicating their less willingness to make friends at work [5,6]; (4) millennials place a higher emphasis on meaningful work. [5]. Due to these differences in work values, millennial workers may shy away from engaging in organizational citizenship behavior (OCB) that requires them to undertake efforts beyond their regular work duties [8]. If millennials place a high emphasis on leisure time and extrinsic rewards, OCB, which is a voluntary action beyond work requirements that provides no formal rewards, is less common among millennials [6,8]. An organization may not formally require its employees to practice OCB. However, OCB often plays a vital role in the effective functioning of the organization [9,10] and is generally linked to the indicators of organizational success [11,12]. Organizational behavior scholars have long believed that successful organizations depend on their employees, who must not only be proficient in completing formal tasks but also engage in involuntary and spontaneous behaviors to support their colleagues and their organizations more broadly [13]. Given the importance of OCB to organizations and the fact that millennials are currently taking over the global workforce, this study emphasizes the necessity of promoting OCB among millennial employees.

Millennial employees respond well to transparent and honest leaders [14,15,16,17]. Ethical leaders care about how employees feel and have a genuine interest in their opinions [18,19]. Millennial employees thrive in an environment where their opinions and views are valued by the leadership [20]. This behavior indicates a positive correlation between millennial employees’ preferred leadership behavior and the ethical leadership style. Long [21] observed that the ethical leadership style is effective in motivating millennial employees. Despite these theoretical foundations, relevant empirical studies are scant [20,22]. Therefore, this study aims to explore the impact of ethical leadership on the OCB of millennial employees.

Brown et al. [23] identified the moral person and the moral manager as the two social learning pillars of ethical leadership. However, only a few studies have empirically explored social learning as a mechanism that explains the connection between ethical leadership and OCB [24]. Existing organizational theories tend to focus on environmental factors and are increasingly becoming “more context sensitive.” Moreover, with the development of positive psychology and positive organizational behavior, the importance of employee well-being has been increasingly emphasized [25,26]. Therefore, this study analyzes the mediating mechanism of ethical climate and affective well-being in the relationship between ethical leadership and OCB from the perspective of social information processing theory and the COR theory.

Additionally, recent research has indicated that the impact of ethical leadership on employees may depend on various individual factors [27]. Thus, the present study contributes to this growing literature by determining whether employee moral identity moderates the impact of ethical leadership on affective well-being.

Small and medium-sized enterprises (SMEs) account for more than 90% of the enterprises in China. Their contribution to the country’s GDP is more than 60% [28]. Unlike large corporations, SMEs typically have a poor market position, which means that their sustainability and growth are heavily reliant on their employees’ willingness to undertake additional efforts beyond the formal limitations of their work activities [29,30]. Leadership behavior is a very important issue for SMEs because, compared to large companies’ formal rules and regulations, leadership behavior exerts a greater influence on employee behavior in SMEs [31]. Effective leadership in SMEs is significant because it serves as a competitive tool in a highly dynamic business environment [32]. Therefore, it is necessary to explore the relationship between ethical leadership and OCB in SMEs.

Managers have been uncertain about managing millennial workers due to their unique values and expectations [33]. This study focuses on the millennial employees of SMEs in China to explore the relationship between ethical leadership and employees’ OCB. It builds a framework to verify the mediating role of ethical climate and job-related affective well-being and the moderating role of moral identity. The results of this study will provide some useful theoretical and practical implications.

## 2. Literature Background and Hypothetical Development

### 2.1. Ethical Leadership and Employees’ OCB

Brown et al. [23] defined ethical leadership as “the demonstration of normatively appropriate conduct through personal actions and interpersonal relationships, and the promotion of such conduct to followers through two-way communication, reinforcement, and decision-making.” They stated that ethical leadership depended on two social learning pillars: the moral person and the moral manager. Ethical leaders are viewed as moral persons because they tend to be trustworthy and honest, take good care of their subordinates, and conduct their personal and professional lives appropriately. They make decisions based on their ethical values and principles. They tend to be fair and concerned about the interests of stakeholders and long-term outcomes [34]. As moral managers, ethical leaders use their positions of authority to promote ethics in the workplace. Ethical managers formulate and communicate ethical standards to their subordinates and use rewards and punishments to ensure they follow these standards [35].

Organ [36] defined OCB as “contributions to the maintenance and enhancement of the social and psychological context that supports task performance.” It refers to the voluntary, discretionary, and altruistic activities conducted by employees outside of their job requirements and for which they may not be paid or rewarded [36,37]. OCB is composed of five aspects, namely, altruism, courtesy, conscientiousness, civic virtue, and sportsmanship. Fassina et al. [38] summarized these five dimensions as follows:

(1) Altruism—Employees express unselfishness by helping other employees in the organization (e.g., helping new employees adapt to the company environment);
(2) Courtesy—Employees inform their colleagues of precautions in advance to prevent them from encountering problems;
(3) Conscientiousness—Employees exhibit behaviors beyond the company’s regulations (e.g., protecting organizational resources);
(4) Civic virtue—Employees demonstrate a positive attitude and responsibility toward company activities (e.g., attending organizational meetings);
(5) Sportsmanship—Employees do not think or act negatively, that is, employees are willing not to complain about coercion and minor inconveniences in the workplace.

Social learning theory [39] can explain the relationship between ethical leadership and its essential outcomes, such as OCB [23]. The theory states that people learn by observing and imitating the values, attitudes, and behaviors of attractive and credible role models. The two most important characteristics of role models are power and status, both of which increase their attractiveness and credibility. According to this theory, when ethical leaders appear as role models in the work environment, employees tend to imitate them. Demonstrating integrity, care, and compassion and supporting others are the characteristics of ethical leadership. Employees learn these behaviors by observing their managers’ behaviors and their reward and punishment behaviors [40]. As ethical leaders, managers strongly influence employee behavior ethically through their assigned identity and power [23]. According to recent research, when an ethical leader demonstrates al-truistic behaviors, such behavior is perceived by subordinates as caring and responsible behaviors. Subsequently, subordinates emulate these behaviors and demonstrate OCB [41]. O’Keefe et al. [42] found that ethical leadership interacts with coworker ethicality to predict employees’ ethical intentions and OCB. Ethical leadership cultivates a sense of moral identity among employees and inspires followers to demonstrate more ethical behaviors, such as increasing their OCB [43]. Arshad et al. [44] found that encouraging ethical leadership and leader–member exchange within organizations promotes OCB among employees. Thus, the following hypothesis was proposed:

**Hypothesis** **1** **(H1).** *Ethical leadership is positively related to employees’ OCB.*

### 2.2. Mediation of Ethical Climate in the Relationship between Ethical Leadership and Employees’ OCB

Based on concepts of ethical philosophy, Victor and Cullen [45] divided ethical climate into three factors: egoism, benevolence, and principle. Egoism is defined as behavior that is primarily motivated by self-interest. Meanwhile, benevolence is related to utilitarianism in that it seeks to achieve the greatest good for the largest number of people through decisions and actions. Lastly, principles refer to making decisions and taking actions in accordance with laws, rules, norms, and procedures, similar to deontology. These three ethical factors constitute the ethical philosophy dimension of the ethical climate framework. One year later, Victor and Cullen [46] divided the ethical climate into five dimensions: “caring” (caring for the well-being of others), “laws and regulations” (whether it violates any laws), “rules” (whether the company’s guidelines and procedures are followed), “instrumentality” (focus on self-interests), and “independence” (adherence to personal ethical beliefs). Therefore, this research conjectures that the ethical climate of an organization includes the normative beliefs and values of ethical issues shared by the organization’s employees. Thus, the ethical climate is similar to moral norms, which are behavioral rules that guide the perception of what is right and wrong within communities and organizations [47,48].

Previous studies regarding ethical leadership stated that ethical leaders act as moral managers. They establish and implement reward and punishment systems to encourage and enforce ethical behaviors among their subordinates [23]. Ethical managers always refer to the reward system, discipline employees who violate ethical standards, and reward employees who demonstrate ethical behavior. In this way, an ethical consensus can be formed within the team [24,49]. Most previous research has shown that the day-to-day behavior of ethical leaders is an important factor that shapes the ethical climate of an organization [50]. According to Aloustani et al. [51], an ethical manager formally talks about ethics and values, explaining how values guide important decisions and the actions of the team. Thus, this study proposed that ethical leadership is positively related to ethical climate.

Social information processing theory [52] is the core theoretical lens used to analyze the possible impact of ethical climate on employees’ OCB. This theory suggests that individuals utilize important cues and information in their surroundings to understand how to behave appropriately in a particular environment. Applying the principles of this theory to the work environment context, employees collect relevant information and clues from their work environments and make/take appropriate decisions/actions accordingly. Therefore, when employees are immersed in an ethical climate and can observe, experience, and interpret various ethical behaviors within the organization, they will subsequently demonstrate ethical behaviors to cater to these organizational practices [48]. Teams with a highly ethical climate may have externally reinforced formal systems that reward ethical behavior or punish unethical behavior. These tangible external rewards will increase employees’ motivation to demonstrate pro-social behavior [24]. Similarly, other team members within a highly ethical climate may also receive rewards for their ethical behavior or be punished for their unethical behavior. The result of these alternative experiences is that team members will learn and behave consistently with the team’s ethical climate, for example, participating in pro-social behaviors such as OCB [51]. Therefore, when employees believe that the climate of their team is ethical, this perception will affect the ethical decision-making and behaviors of the team members [34]. Employees in a highly ethical climate are usually more sensitive to ethical issues, deal with ethical dilemmas in a standardized and appropriate manner, and have a higher ethical awareness [53]. Following the above reasoning, this study proposed that ethical leadership promotes the generation of ethical consensus, and group members, who work together in a common ethical climate and observe similar social implications and cues, are more likely to participate in OCB. Thus, the following hypothesis was proposed:

**Hypothesis** **2** **(H2).** *Ethical climate mediates the relationship between ethical leadership and employees’ OCB.*

### 2.3. Mediation of Affective Well-Being on the Relationship between Ethical Leadership and Employees’ OCB

Job-related affective well-being has been measured as both the positive and the negative feelings experienced by employees in the work environment context [54]. Warr [55] classified the content and intensity of work-related positive and negative emotions on a two-dimensional scale of pleasure and arousal. This scale included six positive feelings (i.e., calm, content, cheerfulness, relaxation, optimism, and enthusiasm) and six negative feelings (i.e., tensed, uneasy, worried, depressed, gloomy, and miserable). According to COR theory [56], people have a fundamental motivation to obtain, retain, and protect what they value. Resources are viewed as “objects, personal characteristics, conditions, or energies that are valued in their own right or that are valued because they act as conduits to the achievements or protection of valued resources.”

The COR theory states that resources, such as ethical leadership, can help employees obtain more resources. This initiates a positive cycle of resources, which can positively impact employee well-being [25]. If supervisors respect their employees’ rights and dignity, care about their welfare, or listen to their concerns and ideas, employees will feel happy or optimistic [35,57]. Conversely, if employees believe that the leader exhibits unethical behavior, such as not intervening or correcting unethical behavior, or failing to communicate their expectations of employees’ ethical behavior and mutual treatment, they will feel angry, worried, and unhappy [41]. The key characteristics of ethical leadership include honesty, trustworthiness, fairness, conscientiousness, and compassion. Given such behavioral traits, ethical leaders can provide job resources by encouraging employees to openly state their worries and will make fair decisions on important issues [58]. Meanwhile, subordinates can receive the help, attention, and emotional care required from their leader, positively affecting their well-being. Ethical leadership is also related to reducing bullying and other workplace misconduct [59].

The COR theory [56] is also particularly valuable for understanding how job-related affective well-being affects OCB. Unlike negative emotions that deplete employees’ resources, job-related affective well-being may help people preserve and develop their personal and work resources. For example, Xu et al. [26] found that employees with high affective well-being tend to set higher goals, motivating them to generate more work resources to achieve these goals. Therefore, this study proposes that affective well-being will promote OCB occurrence and thus increase work resources (e.g., good interpersonal relationships). OCB requires employees to devote their personal and work resources to behaviors that are not formally required by their jobs [60]. Previous research found that participating in OCB may consume a significant amount of human and financial resources. Employees can become exhausted by participating in OCB [61]. Therefore, high-level affective well-being can be viewed as a resource to help employees practice more OCB [26]. Moreover, Hobfoll [62] proposed that “people must invest resources to prevent resource loss, recover from the loss and obtain resources.” Therefore, acquired resources are usually reinvested back into the organization. In line with this, this study expects employees who experience high levels of affective well-being from ethical leadership will repay the benefits of the resources they have obtained by persisting in their OCB. Thus, the following hypothesis was proposed:

**Hypothesis** **3** **(H3).** *Affective well-being mediates the relationship between ethical leadership and employees’ OCB.*

### 2.4. Moderation of Moral Identity

Moral identity is “a self-conception organized around a set of moral traits” representing the degree to which morality is embedded in one’s self-awareness [63]. It refers to “the degree to which a person identifies himself or herself as a moral person” [64]. Fundamentally, moral identity seeks answers to the following questions: ‘‘Am I a moral person or an immoral person?’’ [65]. As a crucial part of an individual’s moral self, moral identity serves as an essential self-regulatory mechanism for ethical behaviors [66]. This is because, based on the motivation of self-consistency, individuals strive to behave in ways that are consistent with how they view themselves, that is, their identity [63,67].

Person–supervisor (P-S) fit refers to the degree of alignment between subordinates and their supervisors in terms of values, personality, work style, lifestyle, and leadership style [68]. In a work environment, subordinates with similar values and work styles as their superiors are more likely to be regarded as reliable people by their superiors and will be entrusted with some responsibilities, which can increase their positive feelings [67]. This means that subordinates whose characteristics align with those of their supervisors can benefit from this alignment. Chuang et al. [67] found that subordinates with a high degree of fit with their supervisors also had more job satisfaction (a measure of job-related well-being). Recent research also found a positive relation between P-S fitness and job satisfaction [69,70,71]. Additionally, Enwereuzor et al. [72] found that a good P-S fit is related to higher mental health levels because the subordinates’ interests are in good alignment with their superiors’ interests, and they get along well. Therefore, there are no confrontations or conflicts between supervisors and subordinates due to different interests. Subordinates who are compatible with their superiors in terms of values, character, work style, lifestyle, and leadership style are more likely to be favored by superiors than subordinates who are not compatible with their superiors [73]. Thus, employees with higher moral identity will have a greater degree of fit with their ethical leaders and subsequently experience more positive feelings than their counterparts with lower moral identity. Thus, the following hypothesis was proposed:

**Hypothesis** **4** **(H4).** *Employees’ moral identity moderates the relationship between ethical leadership and affective well-being such that this relationship is stronger for employees with higher moral identity.*

According to Wen and Ye [74], when the first or second half of the mediation path is moderated by the moderator, the mediating effect will differ at different levels of moderation. The mediation mechanism in the relationship between ethical leadership and OCB is revealed by Hypothesis 3, that is, the “ethical leadership → affective well-being → employees’ OCB” linkage. Furthermore, in Hypothesis 4, this study further theorized that employees with a higher moral identity would experience more job-related affective well-being than employees with a lower moral identity. Thus, this study proposed an integrated effect in which moral identity positively moderates the indirect effect of ethical leadership on employees’ OCB through job-related affective well-being. Thus, the following hypothesis was proposed:

**Hypothesis** **5** **(H5).** *Employees’ moral identity moderates the mediating effect of affective well-being on the relationship between ethical leadership and employees’ OCB. When moral identity is high, this mediating effect is strengthened.*

**Figure 1:**
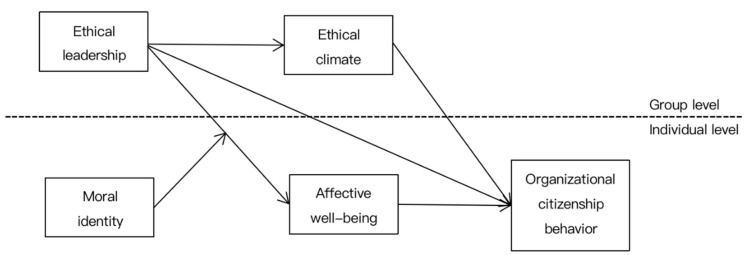
Research model.

Based on the above hypotheses, we designed the research model as shown below (Figure 1).

## 3. Method

### 3.1. Sample and Procedure

The study data were collected from three SMEs in Zhejiang Province and two SMEs in Jiangsu Province, China, from 29 January 2021, to 20 February 2021. For this study, an online questionnaire was designed and distributed to participants via a survey link. To ensure equivalency of meaning, we designed the questionnaire in English, translated it into Chinese, and then back-translated it into English by two bilingual researchers [75]. To minimize common method variance, we sent two different questionnaires to the participants. On the first page of the questionnaire, we explained the purpose of our research and assured all respondents anonymity and confidentiality. After the participants read and agreed with the above content, they could start to fill in the questionnaire. Team leaders were invited to evaluate ethical leadership and ethical climate, whereas subordinates were invited to rate affective well-being, moral identity, and OCB. The respondents were first asked to answer the question “what is your team number?” in the questionnaire. When the questionnaires were sent to the team leaders, they were provided with the different team numbers. They were asked to inform their members of their respective team numbers so that the team number could be used to match the data of team leaders and members. A total of 425 responses were obtained from 67 teams. After removing the invalid and missing questionnaires, 384 valid questionnaires from 61 teams were collected. In this study, a typical work team comprised a team manager and 5–8 subordinates.

SPSS 26.0 (IBM, Armonk, NY, USA) was used to analyze the descriptive statistics based on the basic demographic characteristics of the valid questionnaires. Among the 61 supervisors, 67.2% (*N* = 41) were male and 32.8% (*N* = 20) were female. The ages of the supervisors mainly ranged from 31 to 40 years old, that is, 52.5% (*N* = 32) of the sample, followed by the age range of 41–50 years (22.9%, *N* = 14) and the age range of 20–30 years (21.3%, *N* = 13). Only 3.3% (N = 2) of the supervisors were over 50 years old. Regarding the supervisors’ educational level, 6.1% (*N* = 4) were high school graduates or below, 18.0% (*N* = 11) had college degrees, and 75.4% (*N* = 46) had bachelor’s degrees. Among the 323 subordinates, 59.4% (*N* = 192) were male, and 40.6% (*N* = 131) were female. Regarding the subordinates’ work experience, 88.2% (*N* = 285) had a work experience of 1-3 years, 9.0% (*N* = 29) had a work experience of 4–6 years, and 4.7% (*N* = 9) had a work experience of 7–10 years. Regarding the subordinates’ educational level, 1.9% (*N* = 6) were high school graduates or below, 15.7% (*N* = 51) had college degrees, and 82.4% (*N* = 266) had bachelor’s degrees.

### 3.2. Measures

In this study, a 10-item ethical leadership scale, developed by Brown [23], was adopted to measure the ethical behaviors of leaders. A sample item was “I set an example of how to do things the right way in terms of ethics.” Participants were asked to use a 5-point Likert scale, ranging from 1 = strongly disagree to 5 = strongly agree, to respond to the statements. The reliability of the scale was 0.888. Ethical climate was assessed using Schwepker’s seven-item scale [76]. A sample item was “My work team has a formal, written code of ethics.” Participants were asked to use a 5-point Likert scale, ranging from 1 = strongly disagree to 5 = strongly agree, to respond to the statements. The reliability of this scale was 0.887. Meanwhile, job-related affective well-being was assessed using a 12-item scale developed by Warr [55]. Employees were asked: ‘‘Thinking of the past few weeks, how much time has your job made you feel each of the following’’: relaxed, enthusiastic, cheerful, calm, contented, optimistic, worried, depressed, gloomy, tensed, miserable, and uneasy. Participants were asked to use a 5-point Likert scale, ranging from 1 = always to 5 = never, to respond to the negative statements and a 5-point Likert-type scale, ranging from 1 = never to 5 = always for the positive statements. The scales’ reliability was 0.861. Five items, developed by Zhu [65], were used to measure employees’ moral identity. A sample item was: “I view being an ethical person as an important part of who I am.” Participants used a 5-point Likert scale, ranging from 1 = strongly disagree to 5 = strongly agree, to respond to the statements. The scale’s reliability was 0.881. Finally, OCB was assessed using Williams and Anderson’s 11-item scale [77]. A sample item was “Make constructive suggestions.” Participants were asked to use a 5-point Likert scale, ranging from 1 = strongly disagree to 5 = strongly agree, to respond to the statements. The scale’s reliability was 0.966.

### 3.3. Analysis Strategy

Since subordinates were nested within their supervisors’ teams, this study conducted a multilevel analysis. A supervisor’s ethical leadership and the team’s ethical climate were considered group-level variables, whereas the subordinate’s affective well-being, moral identity, and OCB were considered individual-level variables. Moreover, null model testing was utilized to confirm whether the data were suitable for multilevel analysis. According to the heterogeneity degree classification proposed by Snijder and Bosker [78], an interclass correlation coefficient (ICC) greater than 0.138 indicates a high degree of heterogeneity, and the variation of the dependent variable is not negligible. The null model testing results showed that the ICC of affective well-being was 0.723, indicating that among the reasons for employees’ differences in affective well-being, 72.3% of these differences were caused by differences in the group-level ethical leadership (inter-group variation). Meanwhile, the ICC of OCB was 0.739, which indicates that, among the reasons for employees’ differences in OCB, 73.9% of these differences were caused by differences in the group-level ethical leadership (inter-group variation). These results illustrate the necessity and correctness of the multilevel analysis.

Further, the validity of the constructs and model fit indices were assessed by performing a multilevel confirmatory factor analysis using MPlus 8.3 (MUTHEN & MUTHEN, LA, USA). The model fitness was assessed using the following fit statistics: the chi-square goodness-of-fit to degrees of freedom ratio (χ^2^/df), which must be less than 3; the comparative fit index (CFI) and the Tucker-Lewis fit index (TLI), which must be above 0.9; the root-mean-square error of approximation (RMSEA) and the standardized root-mean-square residual (SRMR), for which values of up to 0.08 are deemed acceptable [79,80,81]. The results showed that χ^2^/DF = 1.280 (<3), CFI = 0.996 (>0.9), TLI = 0.986 (>0.9), RMSEA = 0.029 (<0.08), SRMR within = 0.031 (<0.08), and SRMR between = 0.025 (<0.08). Furthermore, the constructs’ validity and reliability were confirmed using Cronbach’s alpha, composite reliability (CR), average variance extracted (AVE), and maximum shared variance (MSV). Additionally, a multilevel path analysis was performed to test the hypotheses by employing multilevel modeling in MPlus.

Although this study utilized multi-source data to test the study hypotheses, the questionnaire survey was also conducted simultaneously. Thus, this study used Harman’s one-factor test [82] to examine the data for common method variance. The unrotated factor solution showed that one factor explained 22.88% of the variance, which is significantly less than the threshold value of 50%, implying that common method variance was not an issue in this study.

## 4. Data Analysis and Results

The reliability and validity scores for all constructs are reported in Table 1. The Cronbach’s alpha values were satisfactory and exceeded the threshold value of 0.70 [83]. Thus, internal consistency was confirmed for all variables. Likewise, the AVE values were above 0.50, the CR values were above 0.70, and the MSV values were lower than the AVE values [84]; thus, these values were deemed acceptable.

As shown in Table 2, the mean values for the variables were as follows: ethical leadership = 4.036, ethical climate = 4.020, affective well-being = 3.828, OCB = 4.145, and moral identity= 4.256. Furthermore, the standard deviation values for all variables were in the normal range. Additionally, a binary correlation was observed between the research variables in the assumed direction. Therefore, the study data were suitable for further analysis. Meanwhile, the square root of the AVE values, presented along the diagonal, exceeded the value of the correlations, thus proving discriminant validity [85].

Table 3 summarizes the direct effects of the study variables. The regression coefficient of group-level ethical leadership and individual-level OCB was 0.857, *p* < 0.001; ethical leadership and OCB were measured on a 5-point scale. Thus, a one-point increase in ethical leadership is associated with a 0.857-point increase in OCB. Therefore, Hypothesis 1 was supported. The regression coefficient for group-level ethical leadership and group-level ethical climate was 0.806, *p* < 0.001, whereas that for the group-level ethical leadership and individual-level employees’ affective well-being was 0.747, *p* < 0.001. These results indicate that ethical leadership has a positive and statistically significant effect on ethical climate and employees’ affective well-being. Thus, keeping all other variables constant, if ethical leadership increases by one unit, then ethical climate and affective well-being will increase by 0.806 and 0.747 units, respectively.

Table 4 presents a summary of the cross-level mediation effects. Hypothesis 2 posits that the relationship between group-level ethical leadership and individual-level employees’ OCB is mediated by group-level ethical climate. This analysis result indicates a statistically significant, positive mediation effect (EL → EC→ OCB) of 0.220 (95% CI (0.042, 0.482)). Thus, Hypothesis 2 was supported. Hypothesis 3 posits that the relationship between group-level ethical leadership and individual-level employees’ OCB is mediated by individual-level affective well-being. These results indicate a statistically significant, positive mediation effect (EL → AW→ OCB) of 0.239 (95% CI (0.016, 0.463)). Therefore, Hypothesis 3 was supported.

**Figure 2:**
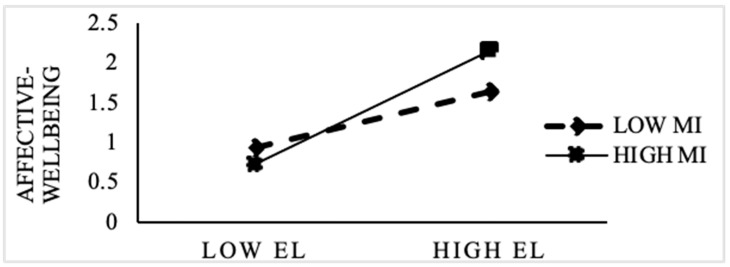
Moderation of moral identity.

Hypothesis 5 proposed that moral identity moderates the indirect effect of ethical leadership on OCB through the mediation of affective well-being. To examine this relationship, this study first tested the interaction between ethical leadership and moral identity and found a significantly positive interaction term (0.142, *p* < 0.001) related to affective well-being (see Table 5). When the moral identity of employees was high, the impact of ethical leadership on employees’ affective well-being was stronger. As shown in Figure 2, compared with low moral identity, in the case of high moral identity, increasing one unit of ethical leadership will promote the more affective well-being of employees. Thus, Hypothesis 4 was supported. Furthermore, Table 5 presents the moderated mediation results obtained using a bootstrap at a confidence interval of 95%. Moral identity played a significant positive moderating role in strengthening the mediation of affective well-being in the relationship between ethical leadership and OCB (0.131, *p* < 0.001). In other words, the indirect effect of ethical leadership on OCB through the mediation of affective well-being was strengthened at a high level of moral identity. Thus, Hypothesis 5 was supported.

## 5. Discussion

The present study aimed to examine the multilevel mechanism of the relationship between group-level ethical leadership and individual-level OCB, mediated by group-level ethical climate and individual-level affective well-being. Moreover, the moderation of individual-level moral identity enriched this framework of leadership research. Based on the empirical analysis, the main results of this study are summarized as follows.

First, group-level ethical leadership has a significant, positive effect on individual-level OCB. Previous research focused on ethical leadership that can promote employees’ ethical behavior and can reduce employees’ unethical behavior [49,86] However, research on the effects of ethical leadership on prosocial behaviors (e.g., OCB) is limited. Following the social learning theory of Bandura [39], we proposed that ethical role modeling comes from leaders who influence the behaviors of employees. Early attempts on this relationship include Tan et al. [41], O’Keefe et al. [42], and Gerpott et al. [43]. However, these studies are all on a single level. Therefore, findings in this study not only align with the existing literature, revealing the positive effect of ethical leadership on OCB, but also explore the relationship between ethical leadership and OCB at multiple levels.

Second, group-level ethical climate partially mediates the relationship between group-level ethical leadership and individual-level OCB. This finding is consistent with the research results of Aloustani et al. [51] and Dinc and Aydemir [87]. Although previous studies have examined the effect of the ethical climate, they have focused on employees’ evaluation of the ethical climate. This research allowed the team leaders to evaluate the team’s ethical climate so that they can make an objective evaluation of the team’s ethical climate from an overall perspective.

Third, individual-level job-related affective well-being partially mediates the relationship between group-level ethical leadership and individual-level OCB. Previous studies have determined that ethical leadership can positively promote employee’s well-being [59,88]. However, studies on the affective well-being of millennials and how affective well-being can promote OCB are limited [89]. Therefore, this study connects ethical leadership and OCB with affective well-being and extends the existing research.

Fourth, moral identity plays a positive moderating role in the relationship between group-level ethical leadership and individual-level job-related affective well-being. Moreover, moral identity positively moderates the mediating effect of individual-level affective well-being in the relationship between group-level ethical leadership and individual-level OCB. When moral identity is high, this mediating effect will be strengthened. Early research showed that the importance of subordinate characteristics needs to be considered in the leadership process [27,86]. In support of this premise, we concluded that the impact of ethical leadership on employees might depend on individual factors.

### 5.1. Theoretical Implications

First, existing organizational theories are increasingly becoming “more context sensitive” [24]. Unlike previous research that studied the relationship between ethical leadership and OCB from a single level [41,42,43], the current research answered the call for context sensitivity by developing a multilevel model of ethical leadership and exploring the relationship between ethical leadership and employees’ OCB. This multilevel model also transforms the current research approach from one that only emphasizes OCB as an outcome of individual-level factors (e.g., organizational identification and psychological empowerment) [90,91] to one that stresses the importance of employees observing and learning from their surrounding environments and emotion-level job-related affective well-being.

Second, this research demonstrates that ethical leadership can directly promote OCB. Ethical leaders are widely believed to be role models for their subordinates and influence their behavior through social learning processes [27,86]. When an ethical leader demonstrates altruistic behaviors, these behaviors are perceived by his/her subordinates as caring and responsible behaviors. Subsequently, subordinates tend to emulate these behaviors and demonstrate more OCB. The results of this research provide empirical evidence for the existing social learning theory.

Third, this study found that a team’s ethical climate significantly affects the relationship between ethical leadership and OCB. This finding is consistent with the findings of Aloustani et al. [51] and Dinc and Aydemir [87]. When leaders become ethical leaders, they can create an environment in which morality is valued [23]. An ethical environment with high ethical standards consistently sends the message that employees must make/take ethical decisions/actions. This message serves as a guideline that directs employees to engage in ethical in-role job performance [24] and extra-role behaviors, such as the OCB proposed in this study. Thus, as a shared consensus among employees of what ethical behavior means to the team, an ethical climate is crucial for shaping employee behavior. This finding confirms the existing social information processing theory [52].

Fourth, with the vigorous development of positive psychology, employee well-being has become a hot topic. However, research on the well-being of millennials has been limited [89]. This study provides evidence that ethical leadership positively impacts the job-related affective well-being of millennials, and “happy” employees are more likely to engage in extra-role behaviors, consistent with Xu et al.’s [26] research results. This finding also provides empirical support for the application of the COR theory. Ethical leadership represents resources offered to subordinates through care, self-belief, and confirmation of the helpfulness of employee’s ideas, which can enhance subordinates’ affective well-being. Subordinates with high levels of affective well-being tend to invest their excess resources back into the organization in the form of OCB.

Finally, ethical leadership improved the job-related affective well-being of subordinates with strong moral identities. This indicates that the impact of ethical leadership on employees may depend on several individual factors. This conclusion addresses Enwereuzor et al.’s [72] call for more researchers to examine P-S fit as a moderator. Subordinates who have similar values and work styles as their superiors will have more affective well-being. Leaders can enhance their subordinates’ well-being based on the quality of their relationships with their subordinates.

### 5.2. Practical Implications

This study provides some practical implications for managers. First, the findings of this study indicate that an ethical leadership style is effective in motivating millennial employees. Ethical leadership behaviors should be evaluated, developed, and rewarded to emphasize the importance of ethical leadership to leaders and encourage them to demonstrate more ethical leadership [24].

Second, compared with large companies, SMEs lack formal rules and regulations [31]. Given the mediating role of ethical climate between ethical leadership and organizational citizenship behavior, each work team should have a clear-written ethics policy specifying expectations for employees and outlining what is and what is not considered acceptable, so that all the members in the team can develop a shared ethical value [87]. In this way, the ethical consciousness of the employees and the ethical climate will be enhanced in the group.

Third, this study proved that employees with high affective well-being could do more organizational citizenship behavior. Kalshoven and Boon [92] found that employees with poor well-being may be less productive, make lower-quality decisions, and be absent from work more frequently. Therefore, for organizations, focusing on improving employee well-being seems important [88]. According to this research, millennials respond well to ethical leadership; therefore, organizations could use ethical leadership, such as power-sharing, caring, and fairness, to enhance employee well-being.

Finally, results of this study reveal that when moral identity is added as a moderating variable, the pure mediating effect of job-related affective well-being on the relationship between ethical leadership and OCB was weakened. This finding supports the importance of considering the characteristics of subordinates in the leadership process [86]. Organizations can strengthen the moral identity of their employees through training [93]. The HR department and each work unit should ensure that their training programs consistently educate employees on the importance of developing an ethical personality and making/taking ethical decisions/actions.

### 5.3. Limitations and Future Directions

Although this study was based on classical theories and provided a statistical basis for the hypotheses, certain limitations should be acknowledged. First, this study utilized a cross-sectional research design, which may have caused some limitations. For example, although this study demonstrates that an ethical climate encourages OCB, the employees’ OCB may improve their team’s ethical climate [24]. Future research should focus on the directionality of the causal relationship between the variables investigated in this study and adopt a longitudinal design to provide more accurate results among the variables. Furthermore, longitudinal studies can also shed light on the long-term effects of observed phenomena (e.g., when leaders consistently demonstrate ethical leadership over time, what impact will this continuous ethical leadership have on the subordinates’ OCB?). Second, future research can adopt experimental designs to test the role of ethical leadership, where participants can be divided into two groups: a treatment group (with a leader high in ethical leadership virtues) and a control group (with a leader low in ethical leadership values). Third, although this study utilized multi-source data with different raters for leader and employee behaviors, the dependent, mediating, and moderating variables were rated by the employees, while the independent variables were rated by the leaders. The data were collected using a self-report survey instrument. Therefore, respondents may not have answered according to their actual situation. Fourth, this study was conducted in a singular culture, China. Thus, further research is needed to extend the research results to other cultures. Chinese society is based on social hierarchies, which implies that Chinese employees may be more influenced by their leaders due to the differences in social status, power, and authority [94]. Moreover, China has a collectivist culture, wherein individuals are more likely to be influenced by the organizational climate. Hence, future research should test for these possible cross-cultural differences [89]. Finally, this research focused on millennial employees from SMEs. Future research can compare the millennial employees of SMEs and large enterprises concurrently to determine whether enterprise size has an impact on employees’ OCB.

## 6. Conclusions

Compared to previous generations, millennials hold unique values, expectations, and attitudes. Due to these differences in work values, millennial workers may be hesitant to participate in OCB. However, OCB often plays a vital role in the effective functioning of the organization and is generally linked to the indicators of organizational success. Since millennials respond well to ethical leadership, we attempted to formulate and investigate the influencing mechanism of ethical leadership on millennial employees’ OCB. A multi-level moderated mediation mechanism encompassing social learning theory, social information processing theory, COR theory, and P-S fit has been used to develop a multi-level model for this research. This study also includes the main effect of ethical leadership, the mediating effect of ethical climate and affective well-being, and the moderating effect of moral identity. Ethical leadership was found to be a significant predictor of ethical climate and affective well-being that ultimately turns into millennial employees’ OCB. Moreover, employees’ moral identity plays an effective moderating role. We strongly emphasize that ethical leaders can have an effective impact on millennial employees. Therefore, organizations should attach importance to the development and training of ethical leadership to deal with the opportunities and challenges brought about by the generational shift in the workforce.

## Figures and Tables

**Table 1:** Reliability and validity of scales.

Variable	Items	Alpha	Factor Loading	CR	AVE	MSV
Ethical leadership	10	0.888	0.652–0.845	0.925	0.554	0.319
Ethical climate	7	0.887	0.603–0.926	0.910	0.597	0.301
Affective well-being	12	0.861	0.614–0.899	0.933	0.541	0.232
OCB	11	0.966	0.703–0.911	0.965	0.718	0.193
Moral identity	5	0.881	0.652–0.882	0.895	0.633	0.318

Notes: Alpha = Cronbach’s alpha, CR = composite reliability, AVE = average variance extracted, MSV = maximum shared variance, OCB = organizational citizenship behavior.

**Table 2:** Correlation matrix of the study variables.

Variable	Mean	SD	1	2	3	4	5
1. Ethical leadership	4.036	0.439	(0.744)				
2. Ethical climate	4.020	0.408	0.549 **	(0.773)			
3.Affective well-being	3.828	0.485	0.482 **	0.355 **	(0.736)		
4. OCB	4.145	0.612	0.373 **	0.324 **	0.237 **	(0.847)	
5. Moral identity	4.256	0.701	0.565 **	0.209 **	0.169 **	0.439 **	(0.796)

Notes: ** *p* < 0.01, Square root of AVE presented along the diagonal, OCB = organizational citizenship behavior.

**Table 3:** Summary of direct effects.

	Estimates	95%CI	Remarks
Group → Group			
EL → EC	0.806 ***	(0.676, 0.935)	
Group → Individual			Supported(1)
EL → OCB	0.857 ***	(0.765, 0.935)	
EL → AW	0.747 ***	(0.578, 0.916)	
EC → OCB	0.273 **	(0.094, 0.360)	
Individual → Individual			
AW → OCB	0.320 *	(0.051, 0.590)	

Notes: * *p* < 0.05, ** *p* < 0.01, *** *p* < 0.001. Bootstrap sample = 1000. CI = confidence interval, EL = ethical leadership, EC = ethical climate, AW = affective well-being, OCB = organizational citizenship behavior.

**Table 4:** Summary of mediation effects.

	Estimates	95%CI	Remarks
Group → Group → Individual			
EL → EC → OCB	0.220 *	(0.042,0.482)	Supported (H2)
Group → Individual → Individual			
EL → AW → OCB	0.239 *	(0.016,0.463)	Supported (H3)

Notes: * *p* < 0.05. Bootstrap sample = 1000. CI = confidence interval, EL = ethical leadership, EC = ethical climate, AW = affective well-being, OCB = organizational citizenship behavior.

**Table 5:** Summary of moderation effects.

	Estimates	95%CI	Remarks
Group * Individual → Individual			
EL * MI→ AW	0.142 ***	(0.115,0.169)	Supported (H4)
Group * Individual → Individual → Individual			
EL * MI → AW → OCB	0.131 ***	(0.093,0.168)	Supported (H5)

Notes: * = interaction term. *** *p* < 0.001. Bootstrap sample = 1000. CI = confidence interval, EL = ethical leadership, EC = ethical climate, AW = affective well-being, OCB = organizational citizenship behavior, MI = moral identity.

## Data Availability

The data used in this research are available on request from the corresponding author. The data are not publicly available due to restrictions, i.e., privacy or ethics.
